# Angiotensin (ang) 1-7 inhibits ang II-induced atrial fibrosis through regulating the interaction of proto-oncogene tyrosine-protein kinase Src (c-Src) and Src homology region 2 domain-containing phosphatase-1 (SHP-1))

**DOI:** 10.1080/21655979.2021.1967035

**Published:** 2021-12-07

**Authors:** Li Lu, Li Cao, Yihao Liu, Yunlin Chen, Jinqi Fan, Yuehui Yin

**Affiliations:** a Department of Critical Care Medicine, University-Town Hospital of Chongqing Medical University, Chongqing, China; b Department of Cardiology, The Second Affiliated Hospital of Chongqing Medical University, Chongqing, China

**Keywords:** Ang-(1-7), ang ii, atrial fibrosis, c-src, shp-1, mapks, collagen

## Abstract

To verify whether Ang-(1-7) produces an antagonistic effect on Ang II-mediated atrial remodeling. Ang II–induced HL-1 cell model and a rat model of Ang II–induced atrial remodeling were constructed and intervened with Ang II Ang-(1-7), AngII +Ang-(1-7), Ang II+ c-Src specific inhibitor (SU6656), and Ang II + Ang-(1-7) + SSG (SHP-1/2 specific inhibitor, stibogluconate), respectively. The systolic blood pressure of the rat caudal artery was detected. And trial fibrosis was detected by Picrosirius red staining and Masson’s trichrome staining. Expressions of transforming growth factor-β (TGF-β), tissue inhibitor of metalloproteinases 1 (TIMP1), Matrix metalloproteinase 2 (MMP-2), connective tissue growth factor (CTGF), galectin-3, α-smooth muscle actin (α-SMA), and collagen I/III were subjected to qPCR and western blot. Furthermore, SHP-1 binding to c-Src was verified by co-immunoprecipitation (Co-IP). Results showed that the expressions of TGF-β, TIMP1, MMP-2, CTGF, α-SMA, galectin-3, and collagen I were increased markedly in the Ang II intervention group, and the expressions of p-ERK1/2, p-Akt, and p-p38MAPK were also increased dramatically. Ang-(1-7) or SU6656 addition could inhibit the action of Ang II factor, thereby minimizing the expressions of the previously described genes and proteins. Simultaneously, SSG supplement reversed the antagonistic effect of Ang-(1-7) on Ang II, and the latter elevated the blood pressure and induced atrial fibrosis in rats. Ang-(1-7) could reverse the changes related to Ang II–induced atrial fibrosis in rats. In conclusion, Ang-(1-7) antagonized Ang II–induced atrial remodeling by regulating SHP-1 and c-Src, thereby affecting the MAPKs/Akt signaling pathway.

## 1 Introduction

Atrial fibrillation represents the commonest sustained arrhythmia that occurs in human beings. It serves as a part of a diverse clinical spectrum of cardiovascular diseases and is correlated to the increased hospital stay, incidence, and mortality [1]. Extensive evidence demonstrates that atrial remodeling, particularly atrial fibrosis, contributes much to the atrial fibrillation substrates, its occurrence, persistence, and progression [1]. It has been noticed that the main effector for heart structural remodeling is the rennin-angiotensin (RAS) axis activation, specifically Ang II. In addition to its vasopressor effect, Ang II, acting via AT1 receptors, stimulates collagen synthesis leading to myocardial interstitial fibrosis [2,3]. The regulatory mechanisms and signaling pathways associated with Ang II–induced atrial remodeling have been investigated widely, but the definite mechanisms have not yet been completely elucidated.

Mitogen-activated protein kinases (MAPKs) are a type of protein kinase that is specific to the amino acids serine/threonine, activated by numerous extracellular stimuli. c-Jun NH2-terminal kinases (JNK), extracellular signal-regulated kinases (ERK1/2), and p38 kinase are three of the principal members [4,5]. Recent studies, as well as this project, have suggested that MAPKs, activated through the process of reversible phosphorylation, can modulate multiple downstream substrates rapidly and efficiently. Meanwhile, it is of great significance for atrial remodeling development when being stimulated by Ang II [6–10]. This study, therefore, focused on stabilizing phosphorylation and dephosphorylation among diverse protein kinases via phosphatases in MAPKs.

As a family of non-receptor tyrosine kinases, c-Src acts via phosphorylation of downstream signaling molecules [6–10]. c-Src kinases ubiquitously present in cells of the heart, endothelial [11], and vascular smooth muscles, and it can mediate multiple signal transduction pathways of myocardial apoptosis, myocardial hypertrophy, and cardiac oxidative stress induced by Ang II [12,13]. Conversely, SHP-1, a redoxsensitive protein tyrosine phosphatase, negatively regulates the activation of protein tyrosine kinase by dephosphorylation. Consequently, the growth and inflammatory signals induced by Ang II can be inhibited [14,15]. Taken together, the dynamic regulation between c-Src and SHP-1 might be the upstream contributor to the MAPKs activation and the potential therapeutic target for the atrial remodeling induced by Ang II.

Ang-(1-7) is recognized as an endogenous polypeptide that primarily derives from Ang II by angiotensin-converting enzyme 2 (ACE2). As the most important endogenous antagonist of the RAS control system, ACE2/Ang-(1-7)/Mas is pivotal to improve the remodeling induced by RAS [16,17]. Recent evidence has indicated a protective effect of Ang-(1-7)/Mas axis in human vascular endothelial cells and renal proximal tubule cells through enhancing SHP-1 activity, and it negatively modulates c-Src activated by Ang II and its downstream signal activation [16,17]. A previous study has revealed that Ang-(1-7) can inhibit collagen production of cardiac fibroblasts in vitro, through activating SHP-1 to counter regulate Ang II–induced phosphorylation of ERK1/2 [7].

We hypothesized that Ang-(1-7) could inhibit Ang II–induced atrial fibrosis through regulating the c-Src/SHP-1 and downstream MAPKs signaling pathway. In this study, to validated the protective role of Ang-(1-7) in atrial fibrosis and gap junctions, we construed a rat model of Ang II–induced atrial remodeling by constant subcutaneous administration and by modulating the phosphorylation balance of the downstream MAPKs.

## 2. Materials and methods

### 2.1 Incubation of cells

HL-1 cells derived from the atrial myocyte line AT-1 of rats were purchased from ATCC. To maintain atrial contractility and stable passage of HL-1 cells, the cells were inoculated in a culture flask containing gelatin/fibronectin, adding with Claycomb medium and 10% fetal bovine serum, 100 μg/mL penicillin, 0.1 mmol/L norepinephrine, and 2 mmol/L L-glutamine. A culture incubator was set at 37°C containing 5% CO_2_, and the solution was refreshed every 24 ~ 48 h [18].

### 2.2 Animal grouping

A total of 30 four-week-old Sprague Dawley (SD) rats were purchased from the Experimental Animal Center of Chongqing Medical University. The animal experiments were strictly followed the ethical requirements of ‘Interim Regulations on the Administration of Experimental Animals of Chongqing Medical University’ and ‘Measures for the Management of Experimental Animal Carcasses and Wastes of Chongqing Medical University’. All animals were classified into a control group, an Ang II (500 ng/kg/min) group, an Ang-(1-7) (500 ng/kg/min) group, an Ang II + Ang-(1-7) group, an Ang II + SU6656, and an Ang II + Ang-(1-7) + SSG group, 5 SD rats per group.

### 2.3 Filling and implantation of delayed-release osmotic capsule

According to the number of animals and drug concentration, drugs were prepared on a super-clean bench, and a quantity of 230 μL was filled into each capsule. A 1 mL empty syringe equipped with a special flat needle was applied to transfer the drug by entering deep into the bottom of the capsule, and slowly infused the drug into the capsule. Liquid exudation at the top of the capsule was the end of the capsule filling. No bubbles were allowed in the capsule, which might affect the penetration rate of the drug. The capsule was capped and kept in normal saline at 37°C for 24 h for thorough activation, allowing the capsule to maintain the desired stable permeation rate being implanted [19]. The SD rats weighing approximately 250 g were selected and intraperitoneally injected with 1% pentobarbital sodium at 0.5 mL/l00 g. Following anesthesia, the animals were shaved, disinfected, and incised a 1-cm-long transverse wound between the scapulars. The hemostatic forceps were deeply inserted into the skin to bluntly dissected a pouch in 1.5 ~ 2 cm depth. The capsule cap was placed inward subcutaneously and adjusted location before the skin was sutured and disinfected. The rats were resuscitated in a warm incubator, closely monitoring the exudation presence from the incision. In case an additional capsule was necessary, another incision was made in the upper edge of the rat buttock before the capsule was implanted. The remaining procedures were identical. The survival of rats, the exudation of incision, and the position of the capsule were observed on the following day. On day 5 after surgery, additional drugs were given mixed with drinking water. Four weeks later, the rats were sacrificed by CO_2_ asphyxiation and the heart was collected for follow-up trials.

### 2.4 Detection of blood pressure in the caudal artery of the rats

The changes in blood pressure were measured weekly by BP-2000. Before measuring, the rats were adaptively measured twice to reduce their tension and irritability. Following being fixed with a clamp, and the blood pressure of the rats was calculated using a photoelectric volume pulse wave recording method when pressure was added to the rat tail. Ten levels of blood pressure were detected each time during three consecutive times to ensure the validity and stability of the data before being exported [20]. Attention should be paid to the changes in systolic blood pressure.

### 2.5 Picrosirius red staining

The paraffin sections were stained with Picrosirius red dye for 1 h. The stained sections were washed in 0.1% acetic acid solution for 30 s. Following being baked or dried at room temperature, the sections were sealed with a water-soluble sealing agent for observation. The fibrotic area was the red stained area [21]. The percentage of positive staining area was analyzed by Image J (National Institutes of Health, USA).

### 2.6 Masson’s trichrome staining

The paraffin sections were stained with Weigert’s iron hematoxylin for 5 min in Masson’s staining kit, washed with running water, differentiated with 1% hydrochloric acid alcohol for 5 s, washed with running water for a few minutes until returning to blue. The sections were stained with ponceau acid fuchsin staining buffer for 5 min, rinsed quickly with distilled water, treated with a phosphomolybdic acid aqueous solution for 3 min, re-stained by aniline blue solution 5 min, treated with 1% glacial acetic acid 1 min, dehydrated and transparent, dried and sealed. Microscopic examination was performed for image acquisition and analysis [22,23]. Collagen volume fraction was analyzed by Image J (National Institutes of Health, USA).

### 2.7 Western blot

Atrial tissue or HL-1 cells were intervened by different intervention groups, added with lysate, and lysed on ice for 30 min, and the supernatant was obtained following centrifugation at 13,800 × g 15 min. The protein concentration was determined by the BCA method, diluted with 5 × loading buffer, and boiled for 5 min at 100°C. SDS-polyacrylamide gel protein was prepared and set at 10% concentration for protein isolation and subsequently transferred to PVDF membrane. Then, 5% TBST-skimmed milk was used for blocking at room temperature for 1 h and incubation lasted overnight at 4°C with primary antibodies. Second antibodies of the corresponding species were supplemented for incubation at room temperature for 1 h after three cycles of membrane washing with TBST, 10 min each time. TBST membrane was washed 3 times, 10 min each time. ECL reaction solution was added, and a Bio-Rad developer was employed for development. Image Lab software was used for quantitative analysis of bands. Antibodies used were as follows: TGF-β (1:500, BM18692, IGEE); TIMP1 (1:1000, BM4959, IGEE), MMP-2 (1:500, BM19080, IGEE), CTGF (1:500, BM11067, IGEE), α-SMA (1:1000, AB7817, Abcam), 3-galectin (1:1000, AB209344, Abcam), p-ERK1/2 (1:500, BMP0472, IGEE), ERK1/2 (1:500, BM4782, IGEE); p-Akt (1:500, BMP0637, IGEE), Akt (1:500, BM16343, IGEE), p-p38MAPK (1:500, BMP0526, IGEE), p38MAPK (1:500, BM0227, IGEE), SHP-1 (1:1000, BM19111, IGEE), c-Src (1:1000, BM19119, IGEE), p-SHP-1 (1:500, BMP0440, IGEE),and p-c-Src (1:500, BMP0452, IGEE).

### 2.8 qPCR detection

The total RNA of atrial tissue or HL-1 cells was extracted by TRIZOL and the total RNA was detected by qPCR after reverse transcription. The reverse transcription reaction was carried out using the TAKARA kit, and the reaction system was 1 μL Reverse Transcriptase, 2.2 μg of RNA, 2 μL of OligodT, 4 μL of dNTP, 4 μL of 5× buffer, 0.5 μL RNAase inhibitor, and up to 20 μL RNAase free ddH2O. The reaction conditions were set at 25°C 5 min, 50°C 15 min, 85°C 5 min, and 4°C 10 min. qPCR experiments were carried out following the instructions of the qPCR kit from Tsingke Biological Technology (Beijing, China). The reaction system was 0.4 μL of forward primer and the same volume of reverse primer, 10 μL of SYBR Green, and 5.2 μL H_2_O. The reaction conditions were set at 50°C 2 min, 95°C 10 min, 95°C 30 sec, and 60°C 30 sec, for 40 cycles. Primers used were as follows: TGF-β-F, 5ʹ-TGAGTGGCTGTCTTTTGACGTC-3ʹ, TGF-β-R, 5ʹ-TTCATGTCATGGATGGTGCC-3ʹ; TIMP1-F, 5ʹ-CGTTTCGTTATTTTTTGTTTTCGGTTTC-3ʹ, TIMP1-R, 5ʹ-CCGAAAACCCCGCCTCG-3ʹ; MMP-2-F, 5ʹ-TCTACTCAGCCAGCACCCTGGA-3ʹ, MMP-2-R, 5ʹ-TGCAGGTCCACGACGGCATCCA-3ʹ; CTGF-F, 5′-AGCCTCAAACTCCAAACACC-3′, CTGF-R, 5′-CAACAGGGATTTGACCAC-3; α-SMA-F, 5′-ATGGCTCCGGGCTCTGTAA-3′, α-SMA-R, 5′-ACAGCCCTGGGAGCATCA-3′; 3-galectin-F, 5′-GGCGCCAGCCCTTACAGCGC-3′, 3-galectin-R, 5′-GGCTTCACCGTGCCCACAAT-3′; GAPDH-F, 5′-CCATGTTCGTCATGGGTGTGA-3′; and GAPDH-R, 5′-CATGGACTGTGGTCATGAGT-3′

### 2.9 Immunohistochemistry

The samples were kept in PBS for 5 min, washed twice, dried the PBS solution around the tissue, added with 3% skimmed milk powder to seal some nonspecific sites, and then put in a 37°C incubator for half an hour. The slide was removed from the incubator, dried the serum on both sides of the slide with absorbent paper, and added with primary antibody, anti-collagen I (1:200, BM1560, IGEE) and anti-collagen III (1:200, BM3795, IGEE), incubated overnight in a refrigerator at 4°C. The slides were taken out of the refrigerator, washed in PBS 3 times, 5 min each time, dried PBS around the tissues, added secondary antibody, and then kept in a 37°C incubator for half an hour. The film was removed from the incubator, placed in PBS, washed 3 times, 5 minutes each time, dried PBS around the tissue, and added DAB color reagent. After counterstaining and mounting, observation was performed under a microscope [24].

### 2.10 Co-IP

Utilizing an appropriate quantity of RIPA lysis buffer, the cells were initially lysed and supplemented with PBS. The working solution with 50% protein A/G-agarose was prepared. and it was subsequently added to the sample at a 1:100 ratio, rocked in a horizontal shaker at 4°C 10 min, and centrifugated at 14,000 g 4°C for 15 min. A new centrifuge tube was ready for supernatant transference, the protein A/G-agarose microspheres were removed simultaneously. The concentration of total protein was subjected to BCA or other methods. A quantity of primary antibodies was supplemented and rocked the antigen-antibody mixture slowly in a shaker at 4°C overnight. The precipitate was collected by centrifugation at 14,000 g, followed by three cycles of washing with precooled washing buffer and then the supernatant was obtained ultimately for western blot assay [25].

### 2.11 Statistical analysis

Statistical analysis was conducted on the experimental data using SPSS 23.0. All obtained data from the experiments were presented as mean ± standard deviation (x ± SD). One-way ANOVA analysis was performed and followed by Tukey tests were adopted for comparison among groups. The values of P less than 0.05 were considered the difference was significant.

## 3 Result

### 3.1 Ang-(1-7) antagonizes II induced atrial fibrosis-related genes expression

In this study, to validated the protective role of Ang-(1-7) in atrial fibrosis and gap junctions, we construed a rat model of Ang II–induced atrial remodeling by constant subcutaneous administration and by modulating the phosphorylation balance of the downstream MAPKs.

**Figure 1.**
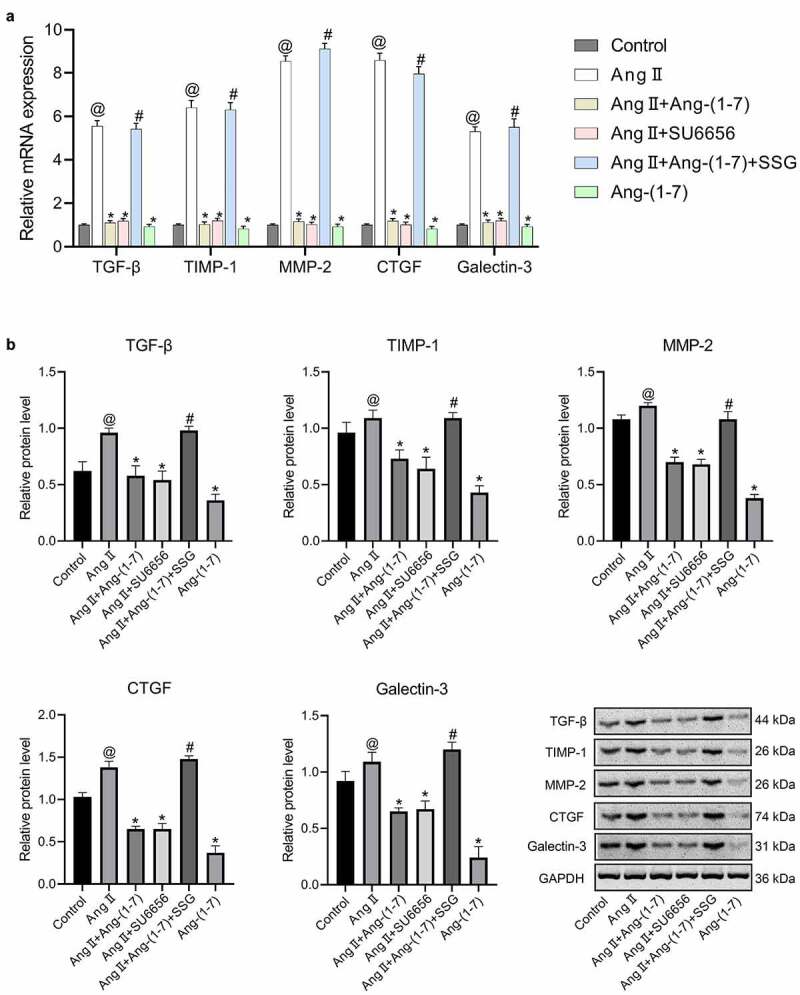
The expressions of atrial fibrosis-related genes and proteins in HL-1 cells treated with Ang-(1-7)/Ang II. (a) The expressions of TGF-β, TIMP-1, MMP-2, CTGF, and galectin-3 genes were determined using qRT-PCR. (b) protein expressions of TGF-β, TIMP-1, MMP-2, CTGF, and galectin-3 were detected by western blot. @ represented p < 0.05 when compared with control; * represented p < 0.05 when compared with Ang II; # represented p < 0.05 when compared with Ang II+Ang-(1-7). Five SD rat samples per group

**Figure 2.**
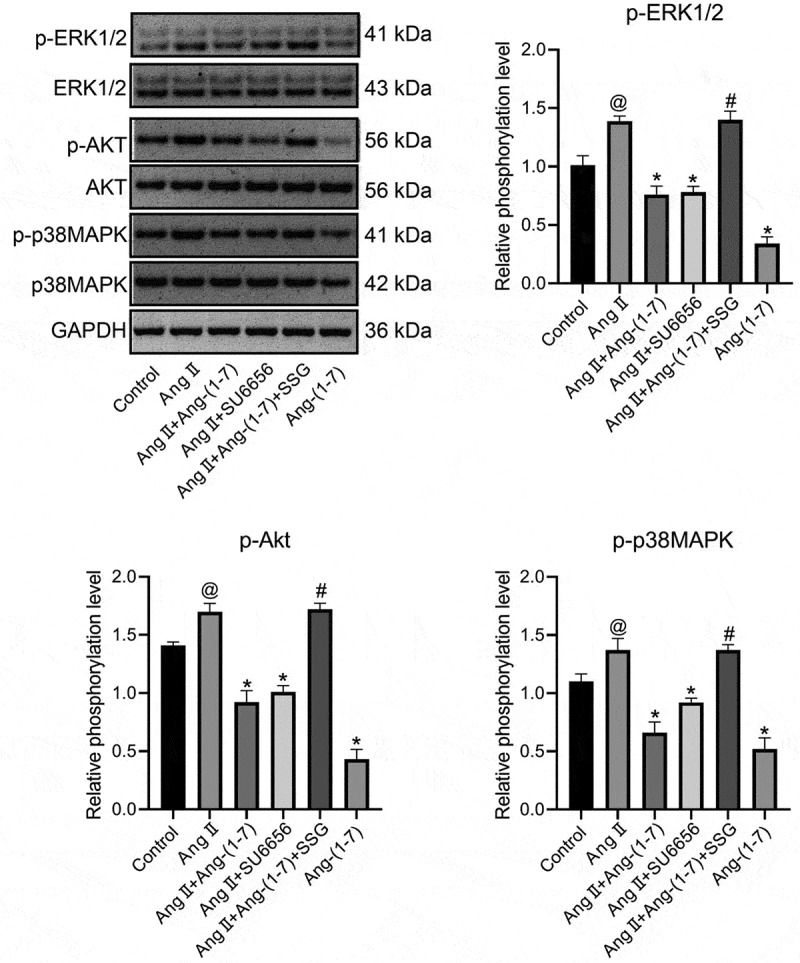
Expression of protein kinase signaling pathway in HL-1 cells treated with Ang-(1-7)/Ang II was determined using western blot. The phosphorylation level is equal to the ratio of phosphorylated proteins to total proteins. @ indicated p < 0.05 in comparison to the control; * indicated p < 0.05 in comparison to Ang II; # indicated p < 0.05 in comparison to Ang II+Ang-(1-7). Five SD rat samples per group

Firstly, to determine the optimal concentration of Ang II, concentrations at 10^−5^ M, 10^−6^ M, and 10^−7^ M were added to HL-1 cells cultured in vitro respectively, and the expressions of fibrosis-related proteins and MAPKs/Akt were detected. The results indicated that following the intervention of Ang II at 10^−^[4] M concentration, the expression of TGF-β was increased substantially (Figure S1). HL-1 cells from the cells cultured in vitro were treated as the control group, Ang II group, Ang-(1-7) group, Ang II + Ang-(1-7) group, Ang II + SU6656 group, and Ang II + Ang-(1-7) + SSG, respectively. The results indicated that following Ang II intervention, the expressions of TGF-β, TIMP1, MMP-2, CTGF, and galectin-3 could be increased markedly (Figure 1(a-b)), and the expressions of p-ERK1/2, p-Akt, and p-p38MAPK were increased dramatically (Figure 2). Ang-(1-7) or SU6656 addition could inhibit Ang II action, meanwhile, the expressions of the previously described genes and proteins were decreased. By adding SSG, Ang-(1-7) reversely antagonized Ang II (Figure 1a-b, 2). The findings indicated a role of Ang-(1-7) in improving atrial fibrosis and Ang II–induced gap junction remodeling by negatively regulating the phosphorylation activation of Ang II and downstream MAPKs/Akt signaling pathway proteins.

### 3.2 Ang-(1-7) antagonizes Ang II-induced systolic blood pressure increase of rat caudal artery

**Figure 3.**
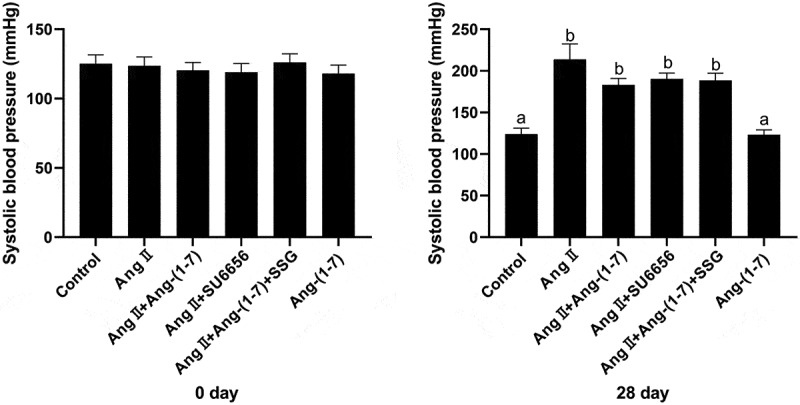
Ang-(1-7) antagonizes Ang II–induced systolic blood pressure increasing of rat caudal artery. significant differences were presented in different letters (p < 0.05). Five SD rat samples per group

To study the effect of Ang-(1-7)/Ang II on systolic blood pressure, the atrial remodeling rat model was construed after continuous subcutaneous injection of Ang II using the osmotic pressure sustained release capsule pump. The effects of each group on the systolic blood pressure of the tail artery were detected on days 0 and 28. The results revealed that Ang II infusion in rats by an osmotic minipump for 28 days increased markedly the systolic blood pressure of the tail artery and Ang-(1-7) infusion alone did not influence systolic blood pressure much. In comparison to the control group, tail artery systolic blood pressure was evidently elevated in the Ang II group, Ang II + Ang-(1-7) group, Ang II + SU6656 group, and Ang II + Ang-(1-7) + SSG group, whereas the difference was not significantly revealed for these groups (Figure 3).

### 3.3 Ang-(1-7) antagonizes ang II-induced atrial fibrosis

**Figure 4.**
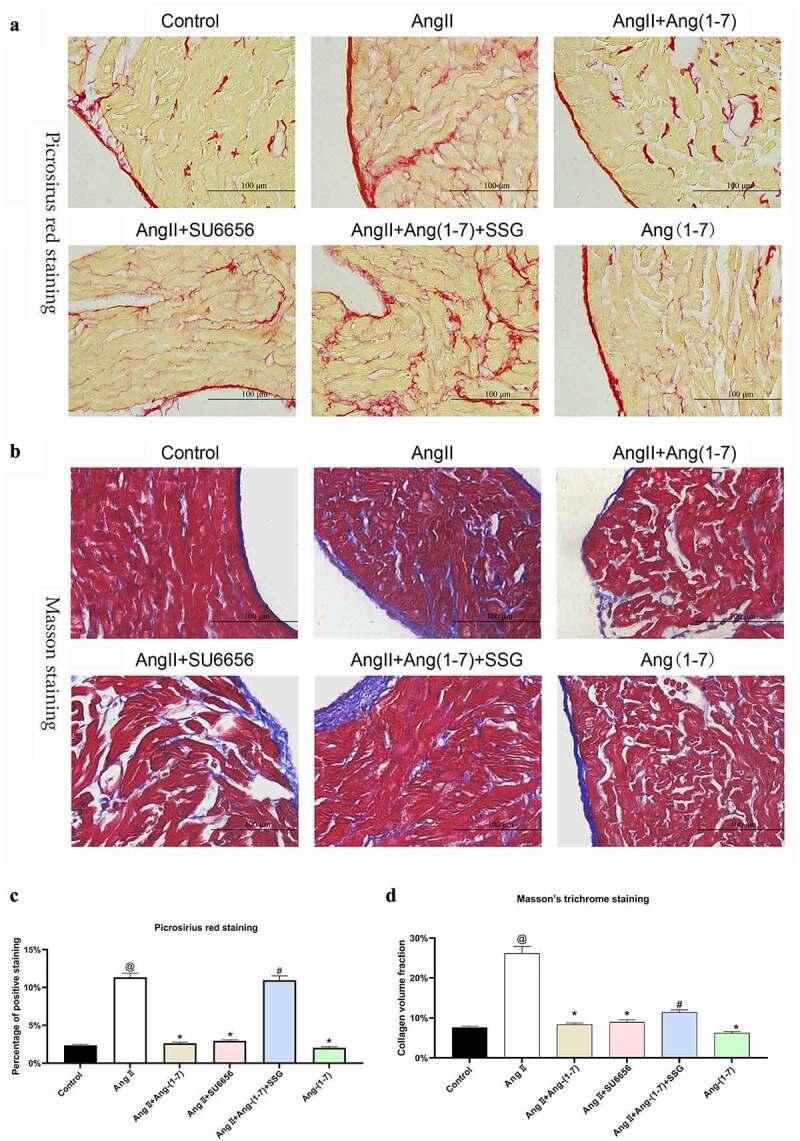
Ang-(1-7) antagonized atrial fibrosis induced by Ang II. (a) picrosirius red staining of atrial sections. (b) masson’s trichrome staining of atrial sections. magnification 400 ×, Bar = 100 μm. @ represented the values of p < 0.05 in comparison to control; * represented the values of p < 0.05 in comparison to Ang II; # represented the values of p < 0.05 in comparison to Ang II+Ang-(1-7). Five SD rat samples per group

Next, the hearts of the rats in each group were taken out, and the morphological changes of the atria were determined using Picrosirius red staining, which indicated the induction of Ang II on atrial fibrosis of the rats. Ang-(1-7) and SU6656 could inhibit Ang II–induced atrial fibrosis, while SSG antagonizing protective effect of Ang-(1-7) (Figure 4(a-c)). By Masson’s Trichrome staining, atrial fibrosis and collagen deposition were further detected. The results indicated that following the induction of Ang II, the gap between the atrial tissues was enlarged, collagen deposition was accumulated, and the degree of fibrosis was increased. After the treatment of Ang-(1-7) and SU6656, fibrosis formation, and collagen deposition could be reduced. Similarly, SSG treatment weakened the antagonistic effect of Ang-(1-7) (Figure 4(b-d)). The changes related to Ang II–induced atrial fibrosis in rats could be substantially reversed after Ang-(1-7) administration, but it failed to decrease Ang II–induced hypertension dramatically.

### 3.4 Ang-(1-7)/Ang II treatment regulates expressions of downstream genes and proteins

**Figure 5.**
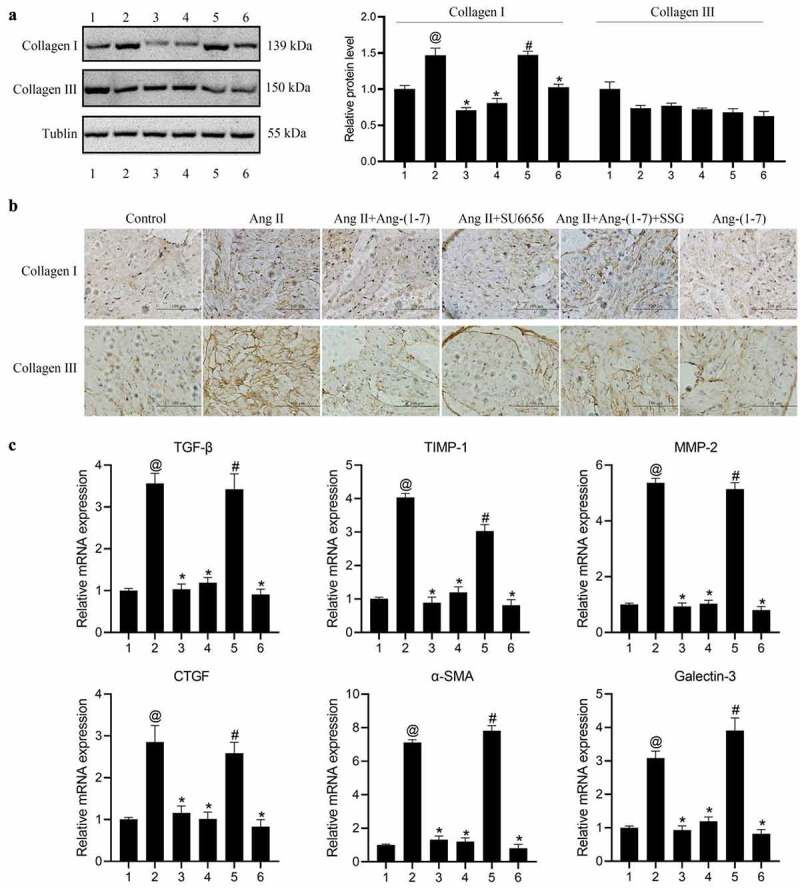
Ang-(1-7)/Ang II treatment regulates expressions of genes and proteins associated with atrial fibrosis. (a) Expressions of Collagen I and III were subjected to western blot. (b) expressions of collagen I and III were determined using immunohistochemistry assays. Bar = 100 μm. (c) expressions of α-SMA, TGF-β, TIMP, MMP-2, CTGF, and galectin-3 were determined using qRT-PCR. @ represented the values of p < 0.05 in comparison to Control; * represented the values of p < 0.05 in comparison to Ang II; # represented the values of p < 0.05 in comparison to Ang II+Ang-(1-7). 1: control; 2: Ang II; 3: Ang II + Ang-(1-7); 4: Ang II + SU6656; 5: Ang II+Ang-(1-7) + SSG; 6: Ang-(1-7). Five SD rat samples per group

To study the effect of Ang-(1-7)/Ang II on the expression of downstream genes and proteins, collagen I/III expression levels were subsequently detected using western blot and immunohistochemical assays. The findings revealed that in accordance with the histological change of fibrosis in atrial tissue, and the level of collagen I was markedly elevated in the Ang II group, which was reversed by Ang-(1-7) or SU6656. Furthermore, the Ang II–induced synthesis of collagen I was reduced as the protective effect of Ang-(1-7) was attenuated by SSG. However, a significant difference in collagen III expressions was not been detected (Figure 5(a-b)).

**Figure 6.**
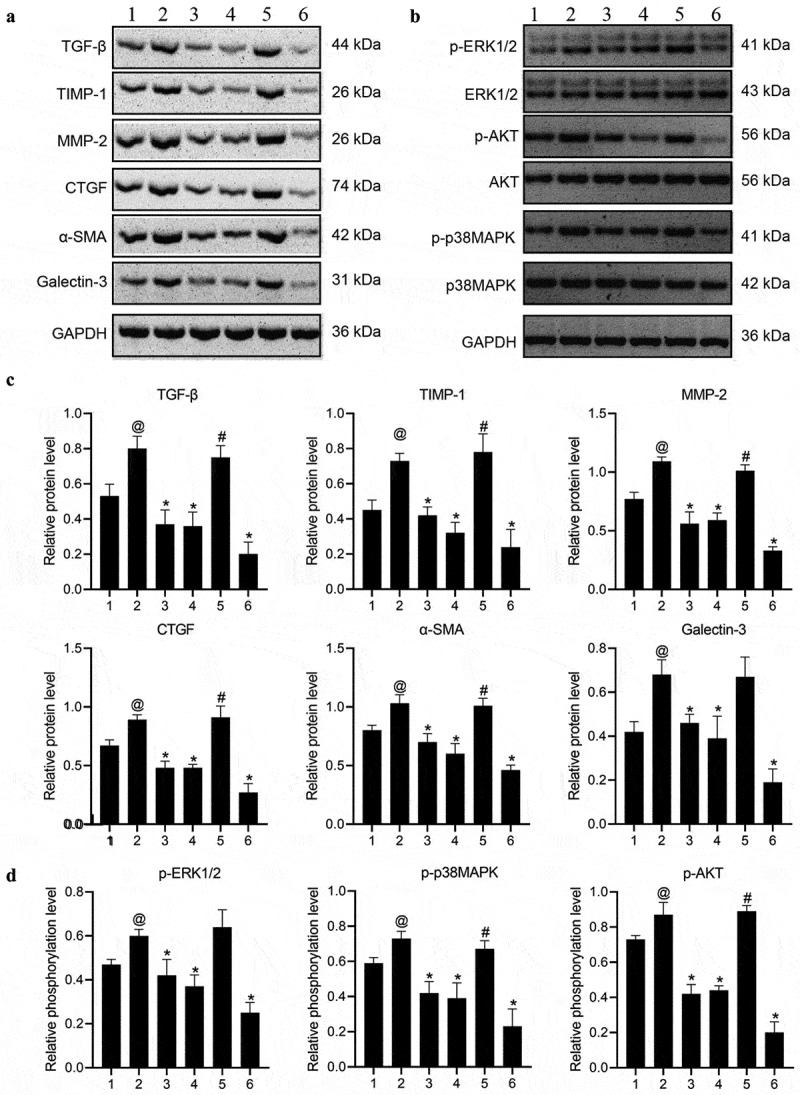
Ang-(1-7)/ang II treatment regulates the expression of atrial fibrosis and protein kinase signaling pathway-related proteins. (a, c) western blot was used to detect expressions of proteins associated with atrial fibrosis. (b, d) western blot was used to determine protein expressions related to the protein kinase signaling pathway. the phosphorylation level is equal to the ratio of phosphorylated proteins to total proteins. @ represented the values of p < 0.05 when compared with control; * represented the values of p < 0.05 when compared with Ang II; # represented the values of p < 0.05 when compared with Ang II+Ang-(1-7). 1: Control; 2: Ang II; 3: Ang II + Ang-(1-7); 4: Ang II + SU6656; 5: Ang II+Ang-(1-7) + SSG; 6: Ang-(1-7). Five SD rat samples per group

The mRNA and protein expressions of TGF-β, TIMP1, MMP-2, CTGF, α-SMA, and galectin-3 were increased markedly revealed in the Ang II intervention group (Figures 5c, 6a, 6c). Meanwhile, the expressions of p-ERK1/2, p-Akt, and p-p38MAPK were elevated dramatically (Figure 6(b-d)). Ang-(1-7) or SU6656 addition could inhibit the effect of Ang II thereby leading to the increase of the expressions of the previously described genes and proteins. By adding SSG, Ang-(1-7) could reversely antagonize Ang II (Figures 5 and 6). This result suggested that Ang II could induce atrial fibrosis in rats by activating downstream protein kinase signaling systems ERK1/2, p38 phosphorylation, and Akt phosphorylation. Ang-(1-7) could improve atrial fibrosis of rats by inhibiting downstream protein kinase signaling systems of ERK1/2, p38 phosphorylation, and Akt phosphorylation. The c-Src inhibitors could inhibit both Ang II–induced c-Src activation and the effect of Ang II on ERK1/2, p38, and Akt signaling pathways, and the SHP-1 inhibitors could also antagonize Ang-(1-7) action on ERK1/2, p38, and Akt signaling pathways induced by Ang II.

### 3.5 SHP-1 binds to c-Src directly

**Figure 7.**
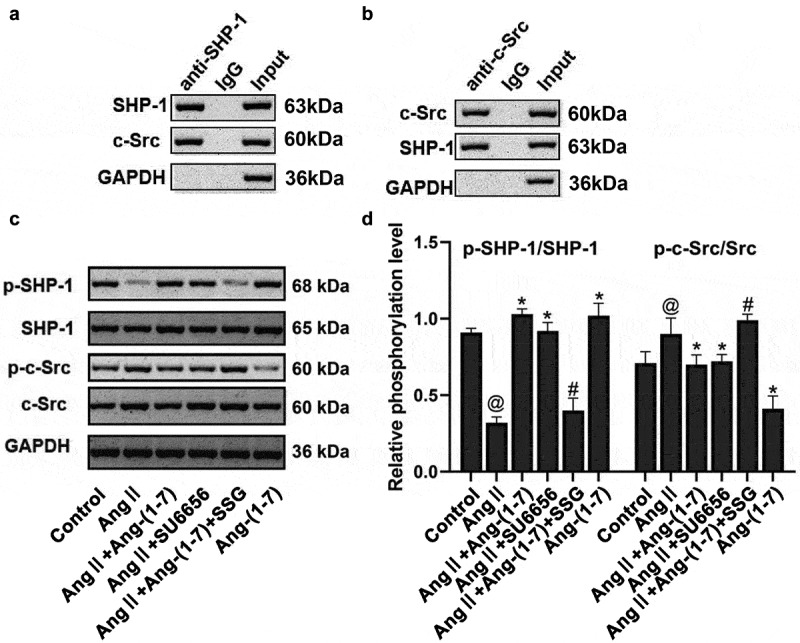
SHP-1 is directly bound to c-Src. (a) Co-IP assay using SHP-1 antibody. (b) Co-IP assay using a c-Src antibody. (c and d) phosphorylation of SHP-1 and c-Src was determined. Ang II could promote c-Src phosphorylation but weaken that of SHP-1. ang-(1-7) antagonized the activation of ang II–induced c-Src through promoting SHP-1 phosphorylation. @ represented the values of p < 0.05 compared with control; * represented the values of p < 0.05 compared with ang II; # represented the values of p < 0.05 compared with Ang II+Ang-(1-7). five SD rat samples per group

To clarify the regulatory mechanism of SHP-1 on c-Src, the Co-IP test revealed that SHP-1 protein could also be detected in the protein complex pulled by SHP-1 antibody (Figure 7(a-b)), indicating that c-Src could directly bind to SHP-1 in cells. Ang II could promote the phosphorylation of c-Src while weakening the phosphorylation of SHP-1. By promoting the phosphorylation of SHP-1, Ang-(1-7) antagonized Ang II–induced activation of c-Src, while the mutual binding between c-Src and SHP-1 played a regulatory role during this process (Figure 7(c-d)). It was speculated that by regulating SHP-1 and c-Src, Ang-(1-7) activated the activity of proteins related to the protein kinase signaling pathway (ERK1/2, Akt, and p38MAPK) and downregulated the expressions of TGF-β/MMP-2/TIMP-1/collagen I, to antagonize AngII induced atrial fibrosis remodeling, thereby ultimately play a protective role in atrial fibrillation.

## 4 Discussion

Ang II functions as a main component of the angiotensin converting enzyme (ACE)/Ang II axis [26]. It can affect the proliferation and phenotypic changes of cardiomyocytes by mediating the expression of TGF-β1 [27]. The activation of the renin-angiotensin-aldosterone system leads to an increase in Ang II expression, promotes atrial fibrosis remodeling and electrical remodeling, and increases the risks of atrial fibrillation incidence. As an essential antagonistic system of the ACE/Ang II axis, Ang-(1-7) can upregulate heart-related channel proteins and minimize AF-associated incidence and duration [8,16]. This work verified the antagonistic action of Ang-(1-7) on Ang II–induced atrial remodeling in vivo and the role of dynamic balance between SHP-1 and Src. Ang II Ang-(1-7), Ang II + Ang-(1-7), Ang II + SU6656 and Ang II + Ang-(1-7) + SSG were administered to the rat model in vivo, and the HL-1 cell model cultured in vitro, respectively, for intervention. The results indicated a role of Ang-(1-7) in reversing changes associated with Ang II–induced atrial fibrosis in rats, but it failed to decline Ang II–induced high blood pressure dramatically. The expressions of TGF-β, TIMP1, MMP-2, CTGF, α-SMA, galectin-3, and collagen I in the Ang II intervention group, could be increased markedly, and expressions of p-ERK1/2, p-Akt, and p-p38MAPK were also increased dramatically. Ang-(1-7) or SU6656 addition could inhibit Ang II actions, and expressions of the previously described genes and proteins were simultaneously decreased. Moreover, c-Src directly binds to SHP-1 was also confirmed via the Co-IP assay.

Ang II acts as a principal effector molecule of RAS, its overactivation exerts a key role in atrial fibrillation occurrence and maintenance [27]. c-Src exists widely in cardiomyocytes and participates in Ang II signal transduction which was consequently activated due to Ang II [15]. Studies have confirmed that RAS inhibitors can reduce the load of atrial fibrillation to some extent, retard disease progression to persistent atrial fibrillation [1,26]. ACE2/Ang-(1-7)/Mas, being an important endogenous regulatory axis of RAS, generates a vital influence on negatively regulating Ang II mediated myocardial remodeling [1,26]. In the previous study of cardiac fibroblast in rats, Ang-(1-7) has been reported to limit ERK activation induced by Ang II through SHP-1 activation, thereby inhibiting the production of TGF-β and collagen, presenting the effect of anti-myocardial fibrosis [28,29]. The current research confirmed hypertension caused by Ang II (RASS activation) and induced atrial fibrosis rather than ventricular fibrosis in rats. Atrial fibrosis involved the changes of collagen fiber regulatory factors TGF-β/MMP-2/TIMP1, and the expression of collagen I increased predominantly. Ang-(1-7) reversely altered Ang II–induced atrial fibrosis in rats, but it failed to decrease Ang II–induced hypertension dramatically.

Protein phosphorylation is critical to the activation of protein functions. The phosphorylation of key proteins in the MAPK pathway marks the activation and function of the signal pathway [28,29]. Previous studies have suggested that the Ang-(1-7)/Mas axis may negatively mediate c-Src activation including Ang II-induced downstream signals via enhancing SHP-1 activity of renal proximal convoluted tubule cells, thereby realizing the protective effect [29–31,32]. In this study, Ang-(1-7) indicated a role in improving atrial fibrosis in rats by inhibiting downstream protein kinase signaling systems ERK1/2, p38 phosphorylation, and Akt phosphorylation. c-Src inhibitors could not only restrain c-Src activation induced by Ang II, but also block Ang II action on ERK1/2, p38, and Akt signaling pathways, and SHP-1 inhibitors could also antagonize Ang-(1-7) effects on ERK1/2, p38, and Akt signaling pathways induced by Ang II [32].

Ang II could promote c-Src phosphorylation while weakening SHP-1 phosphorylation. Ang-(1-7) antagonized Ang II–induced c-Src activation by promoting SHP-1 phosphorylation [29–31]. Moreover, c-Src bound to SHP-1, and this combination played a regulatory role. It was, therefore, speculated that Ang-(1-7) activated SHP-1, thereby inhibiting Ang II-activated c-Src, and then restraining activation of ERK1/2, p38, and Akt phosphorylation. The expressions of TGF-β, MMP-2, and TIMP1 were subsequently reduced, whereas the expression of collagen I was decreased, thereby blocking atrial fibrosis. This study further revealed the regulatory interrelationship between Ang-(1-7) and Ang II and the molecular regulatory mechanisms as well. It is expected to lay a research foundation for clinical reference in future atrial fibrillation treatment.

## 5. Conclusions

Ang-(1-7) antagonized Ang II–induced atrial remodeling by regulating SHP-1 and c-Src, thereby affecting the MAPKs/Akt signaling pathway and regulating the expressions of TGF-β, MMP-2, TIMP1, and collagen I.

## Supplementary Material

Supplemental Material
