# Supplementary material for: Angiotensin (ang) 1-7 inhibits ang II-induced atrial fibrosis through regulating the interaction of proto-oncogene tyrosine-protein kinase Src (c-Src) and Src homology region 2 domain-containing phosphatase-1 (SHP-1))
Source: Bioengineered. 2021 Dec 7;12(2):10823–36. doi: 10.1080/21655979.2021.1967035 (PMC8809921; doi:10.1080/21655979.2021.1967035)
Supplement: Supplemental Material [file KBIE_A_1967035_SM5290.zip › sup.pdf]

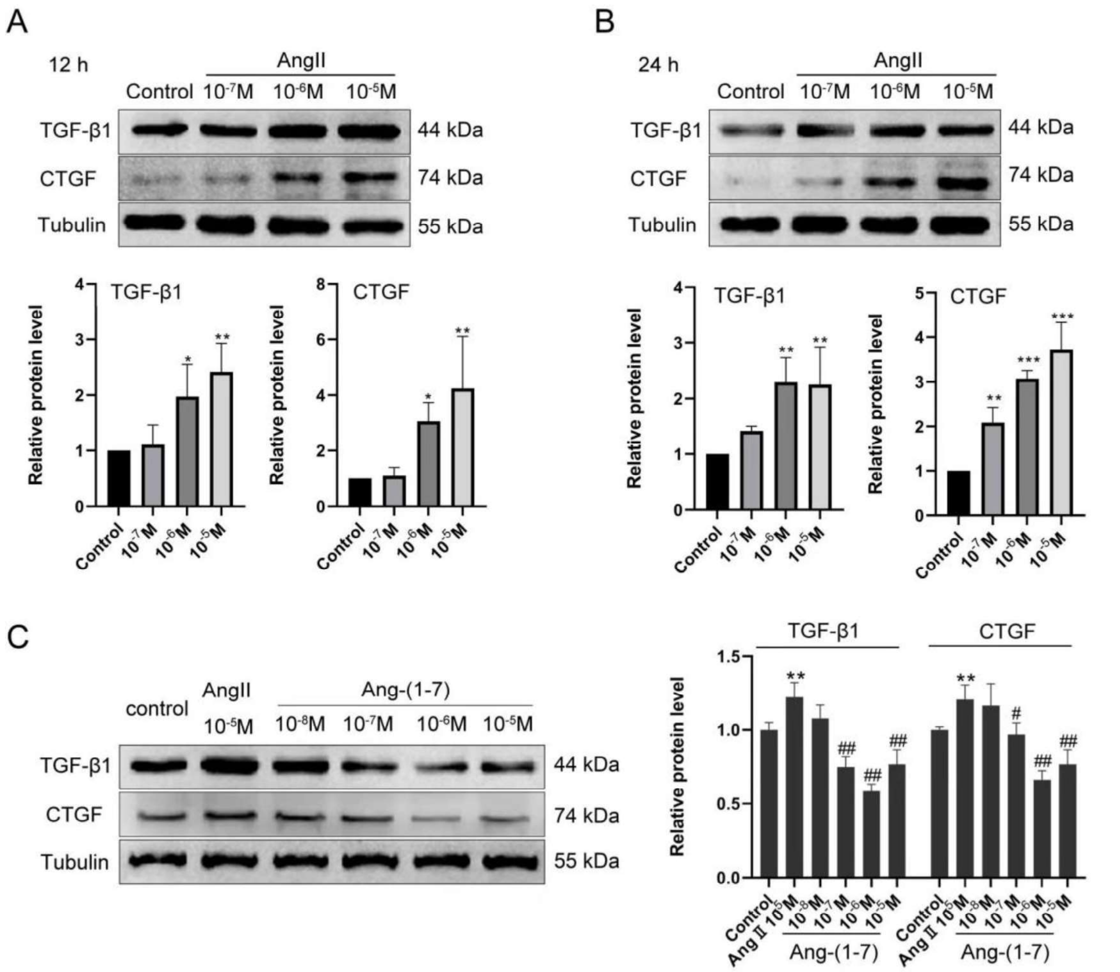

Figure S1. The drug concentration and treatment time of Ang II and Ang-(1-7) intervening in HL-1 cells. A: HL-1 cells were treated with Ang II 10^{-7}, 10^{-6}, and 10^{-5} mol/L for 12 h. Western blot was used to detect the expression of fibrosis-related indicators TGF-β and CTGF. B: HL-1 cells were treated with Ang II 10^{-7}, 10^{-6}, and 10^{-5} mol/L for 24 h. Western blot was used to detect the expressions of TGF-β and CTGF. C: HL-1 cells were pretreated with Ang-(1-7) 10^{-8}, 10^{-7}, 10^{-6}, and 10^{-5} mol/L for 30 min, and then treated with Ang II 10^{-5} mol/L for 24 h. *P <0.05, **P <0.01, ***P <0.001, compared with Control. #P <0.05, ##P <0.01, compared with the Ang II group. Five SD rat samples per group.
